# Supplementary material for: Enhancing Preoperative Outcome Prediction: A Comparative Retrospective Case–Control Study on Machine Learning versus the International Esodata Study Group Risk Model for Predicting 90-Day Mortality in Oncologic Esophagectomy
Source: Cancers (Basel). 2024 Aug 29;16(17):3000. doi: 10.3390/cancers16173000 (PMC11394558; doi:10.3390/cancers16173000)

Supplementary material

Table S1: Categorical parameters.

**Catergorical parameters**	**Value**
Sex	female
	male
Age at surgery	<= 40
	41-50
	51-60
	61-70
	71-80
	>80
Body mass index (BMI)	< 18.5
	18.5-24.9
	25-29.9
	>=30
	< 18.5
ASA-Score	ASA 1
	ASA 2
	ASA 3
	ASA 4
	ASA 5
Stages of preoperative renal function	Stage 1: GFR >= 90 ml/min
	Stage 2: GFR 60 - 89 ml/min
	Stage 3: GFR 30 - 59 ml/min

	Stage 4: GFR 15 - 29 ml/min
	Stage 5: GFR < 15 ml/min
ECOG (Eastern Cooperative Oncology Group) stage	0
	1
	2
Preoperative tumor status (Tpre)	cT0
	cT1
	cT2
	cT3
	cT4
	cTis
	cTx
Preoperative nodal status (Npre)	cN0
	cN1
	cN2
	cN3
	cNX
Preoperative treatment	Radiochemotherapy
	Chemotherapy
	Radiotherapy
	None
Histology	Adenocarcinoma
	Squamous-cell carcinoma
	Other
Tumor location	cervical
	upper thoracic section
	mid thoracic section

	lower thoracic section
	GEJ (gastro-esopgageal junction)

Table S2: Preoperative lab values within 28 days before surgery

**Preoperative (PreOP) laboratory values**	**Unit**
PreOP sodium	mmol/l
PreOP potassium	mmol/l
PreOP urea	mg/dl
PreOP thyroid stimulating hormone (TSH)	mU/l
PreOP alanine aminotransferase (ALAT)	U/l
PreOP aspartate aminotransferase (ASAT)	U/l
PreOP bilirubin	mg/dl
PreOP gamma-glutamyltransferase (GGT)	U/l
PreOP lipase	U/l
PreOP c-reactive protein (CRP)	mg/l
PreOP hemoglobin	g/dl
PreOP white blood cell count (WBC)	/nl
PreOP platelets count	/nl
PreOP hematokrit	
PreOP prothrombin time (PT)	%
PreOP international normalised ratio (INR)	
PreOP activated partial thromboplastin time (aPTT)	seconds
PreOP red blood cell count (RBC	/pl

Table S3: Comorbidities according to the revised Charlson Comorbidity Score

Comorbidities	
Myocardial infarction	yes/no
Peripheral vascular disease	yes/no
Dementia	yes/no
Chronic pulmonary disease	yes/no
Rheumatic disease	yes/no
Peptic ulcer disease	yes/no
Liver disease mild	yes/no
Diabetes without chronic complications	yes/no
Diabetes with chronic complications	yes/no
Hemiplegia or paraplegia	yes/no
Any malignancy	yes/no
Liver disease moderate to severe	yes/no
Renal disease	yes/no
Metastatic solid tumor	yes/no
Charlson Comorbidity Score	numeric

Table S4: IESG score distribution of the entire study cohort.

IESG score	Alive	Deceased	Total	% Mortality
**>= -10**			0	
**-9**			0	
**-8**	1	0	1	0,0
**-7**	1	0	1	0,0
**-6**	2	2	4	50,0
**-5**	6	1	7	14,3
**-4**	16	3	19	15,8

**-3**	20	3	23	13,0
**-2**	25	0	25	0,0
**-1**	78	6	84	7,1
**0**	124	1	125	0,8
**1**	150	9	159	5,7
**2**	68	6	74	8,1
**3**	23	1	24	4,2
**4**	6	0	6	0,0
**5**			0	
**Sum**	520	32	552	

Table S5: AUROC, AUPRC and MCC after internal validation.

Classifier	AUROC mean, 95% CI (low-high)	AUPRC mean, 95% CI (low-high)	MCC mean, 95% CI (low-high)
	**90-day mortality**		
Decision Tree	0.57 (0.54-0.59)	0.14 (0.11-0.18)	0.17 (0.13-0.20)
Gradient Boosting	0.72 (0.69-0.74)	0.18 (0.15-0.20)	0.15 (0.12-0.19)
Linear Support Vector Machine	0.69 (0.66-0.71)	0.15 (0.13-0.17)	0.23 (0.19-0.27)
Logistic Regression	0.75 (0.73-0.78)	0.19 (0.17-0.21)	0.37 (0.34-0.39)
Neural network	0.69 (0.67-0.71)	0.15 (0.13-0.17)	0.24 (0.20-0.27)
Random forest	0.70 (0.68-0.73)	0.15 (0.13-0.17)	0.30 (0.27-0.32)
Support Vector Machine	0.72 (0.69-0,74)	0.20 (0.17-0.23)	0.27 (0.24-0.31)
	**30-day mortality**		
Decision Tree	0.49 (0.46-0.51)	0.06 (0.03-0.09)	0.03 (0.02-0.05)
Gradient Boosting	0.49 (0.46-0.53)	0.04 (0.03-0.06)	0.01 (0.00-0.03)
Linear Support Vector Machine	0.49 (0.45-0.53)	0.04 (0.03-0.06)	0.02 (0.00-0.05)
Logistic Regression	0.50 (0.47-0.54)	0.04 (0.02-0.05)	0.11 (0.08-0.13)
Neural network	0.49 (0.45-0.54)	0.05 (0.03-0.07)	0.07 (0.05-0.09)
Random forest	0.48 (0.45-0.51)	0.03 (0.02-0.04)	0.10 (0.08-0.12)
Support Vector Machine	0.47 (0.44-0.51)	0.04 (0.03-0.05)	0.01 (0.00-0.03)

Table S6: Difference in metric performance of all classifiers between training and validation cohort. One-sided Mann-Whitney U test to evaluate the difference of every classifier: * p < 0.05, significant; ** p < 0.01, highly significant.

Classifiers	p-value	significance level
	**AUROC**	
DecisionTreeClassifier	p-value < 0.05	*
RandomForestClassifier	p-value < 0.01	**
GradientBoostingClassifier	p-value < 0.01	**
LogisticRegression	p-value < 0.01	**
SVC	p-value < 0.01	**
LinearSVC	p-value < 0.01	**
MLPClassifier	p-value < 0.01	**
	**AUPRC**	
DecisionTreeClassifier	p-value < 0.01	**
RandomForestClassifier	p-value < 0.01	**
GradientBoostingClassifier	p-value < 0.01	**
LogisticRegression	p-value < 0.01	**
SVC	p-value < 0.05	*
LinearSVC	p-value = 0.68	
MLPClassifier	p-value < 0.01	**

Table S7: Complete model performance metrics after external validation

**Classifier**	**Average precision**	**Balanced accuracy**	**F1 score**	**MCC**	**AUPRC**	**Precision**	**Recall**	**AUROC**
		90-day mortality						
DummyClassifier	0.04	0.50	0.08	0.00	0.52	0.04	1.00	0.50
DecisionTreeClassifier	0.04	0.50	0.08	0.00	0.03	0.04	1.00	0.45
RandomForestClassifier	0.06	0.60	0.13	0.09	0.05	0.08	0.43	0.50
GradientBoostingClassifier	0.08	0.50	0.08	0.00	0.06	0.04	1.00	0.64
LogisticRegression	0.07	0.56	0.17	0.14	0.05	0.20	0.14	0.51
SVC	0.04	0.55	0.15	0.12	0.03	0.17	0.14	0.38
LinearSVC	0.06	0.55	0.13	0.08	0.05	0.11	0.14	0.49
MLPClassifier	0.05	0.50	0.08	0.00	0.04	0.04	1.00	0.46
		30-day mortality						
DummyClassifier	0.01	0.50	0.01	0.00	0.50	0.01	1.00	0.50
DecisionTreeClassifier	0.06	0.95	0.11	0.22	0.03	0.06	1.00	0.94
RandomForestClassifier	0.01	0.59	0.02	0.04	0.00	0.01	1.00	0.26
GradientBoostingClassifier	0.01	0.50	0.01	0.00	0.00	0.01	1.00	0.23
LogisticRegression	0.01	0.77	0.03	0.08	0.01	0.01	1.00	0.55
SVC	0.02	0.47	0.00	-0.02	0.01	0.00	0.00	0.60
LinearSVC	0.01	0.46	0.00	-0.02	0.01	0.00	0.00	0.56
MLPClassifier	0.01	0.50	0.01	0.00	0.01	0.01	1.00	0.40

Figure S1: Schema of the model development

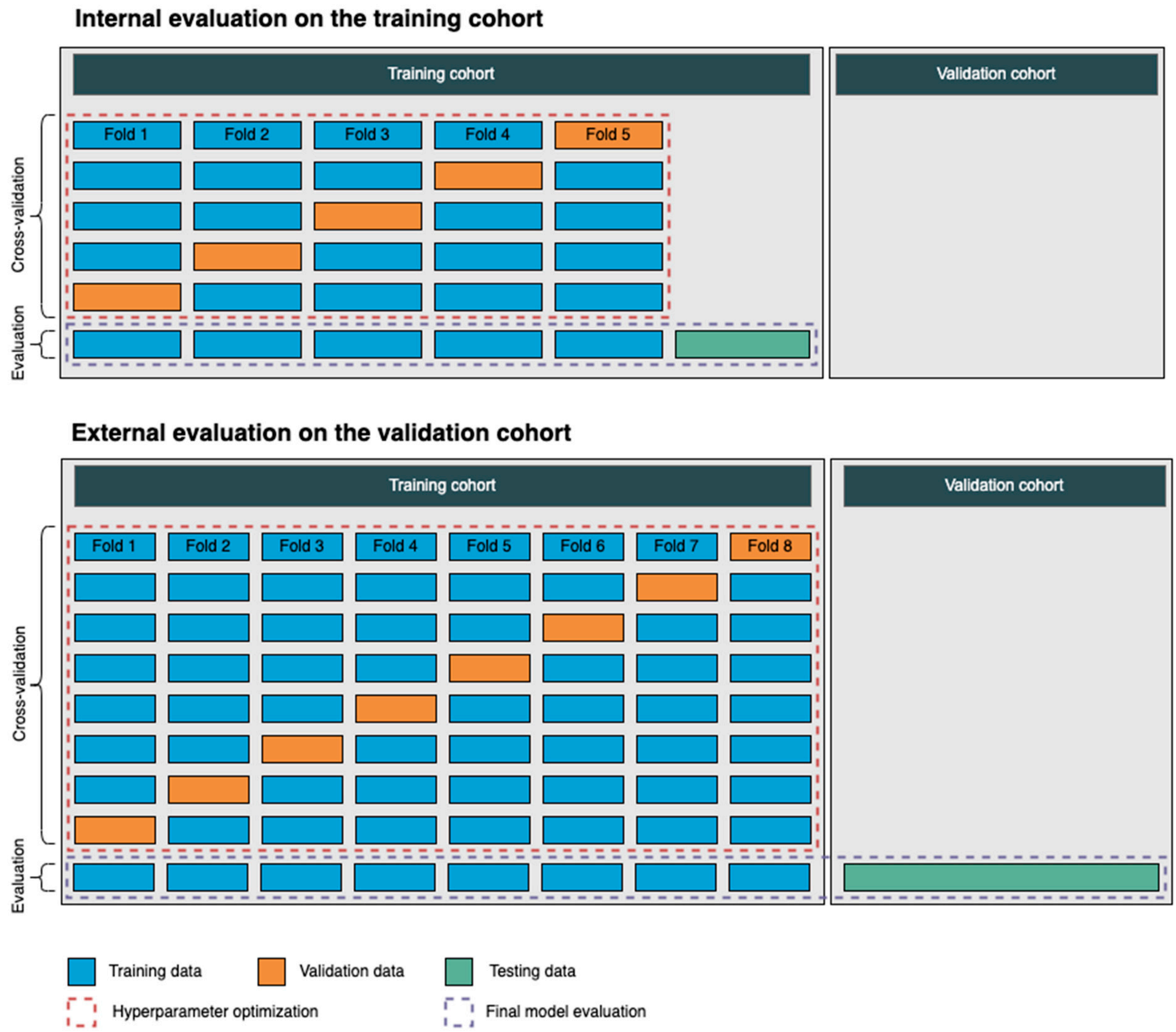

Figure S2: ROC (a) and PRC (b) of all classifiers after external validation

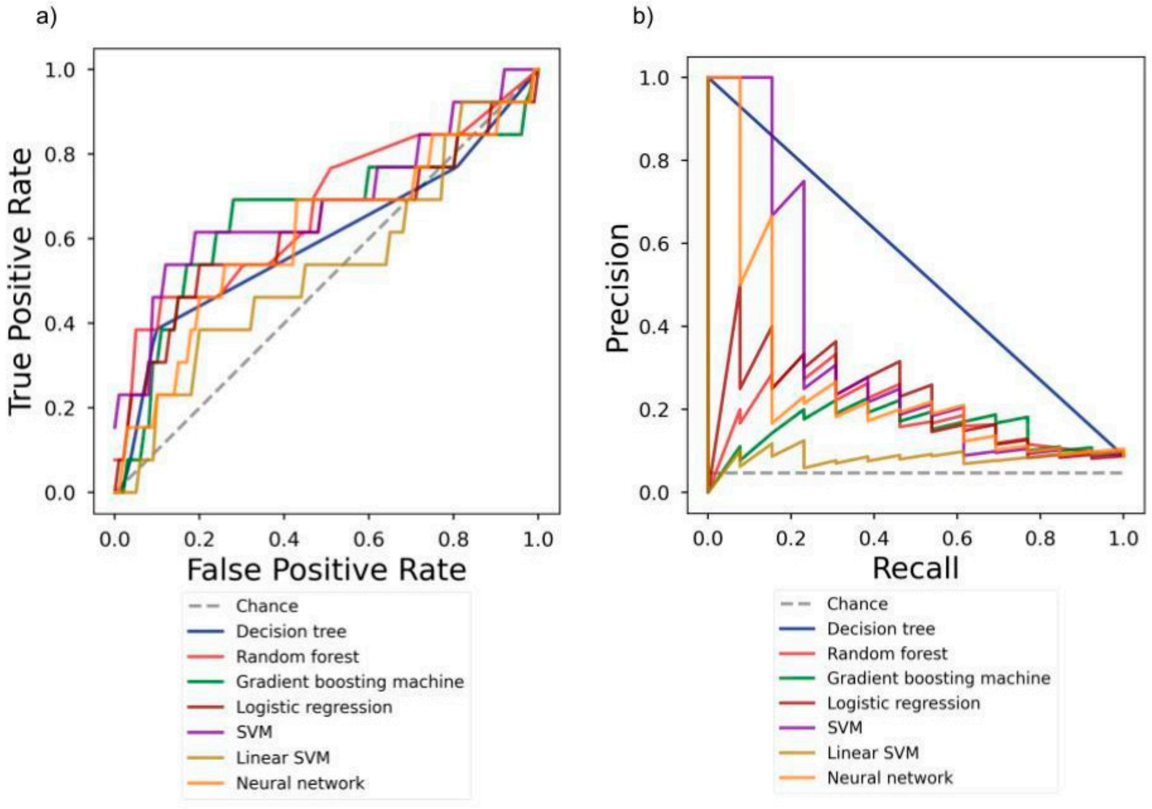

Figure S3: Normalized top ten Shap values Decision Tree, descending

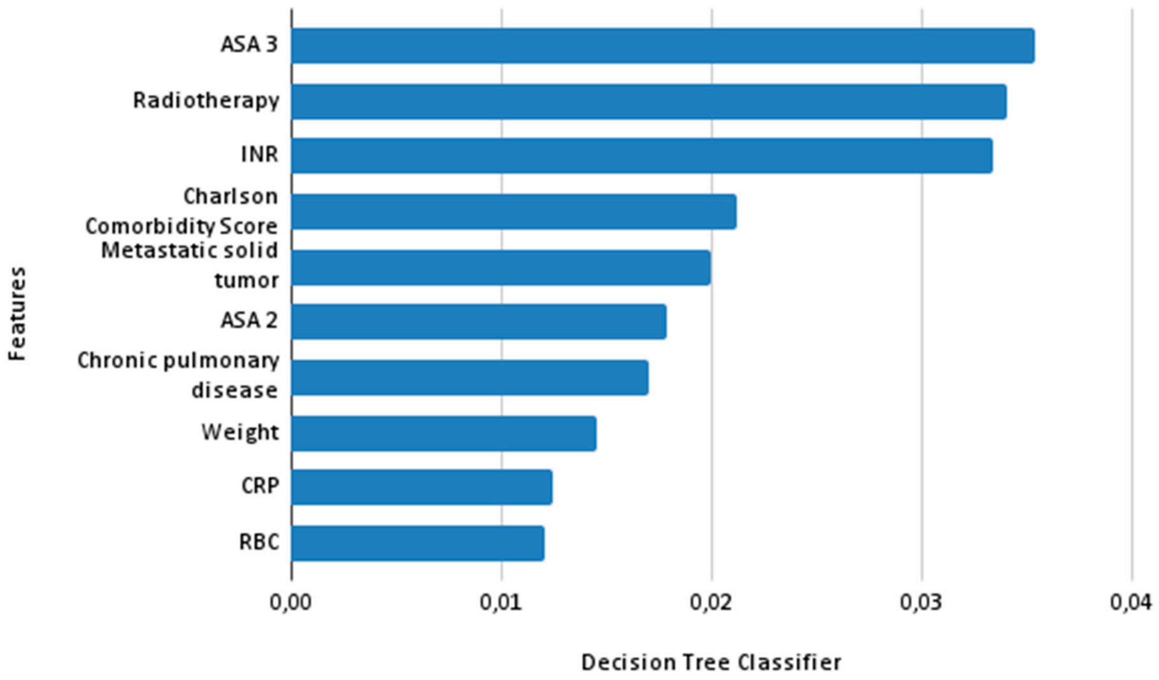

Figure S4: Normalized top ten Shap values Gradient Boosting Classifier, descending

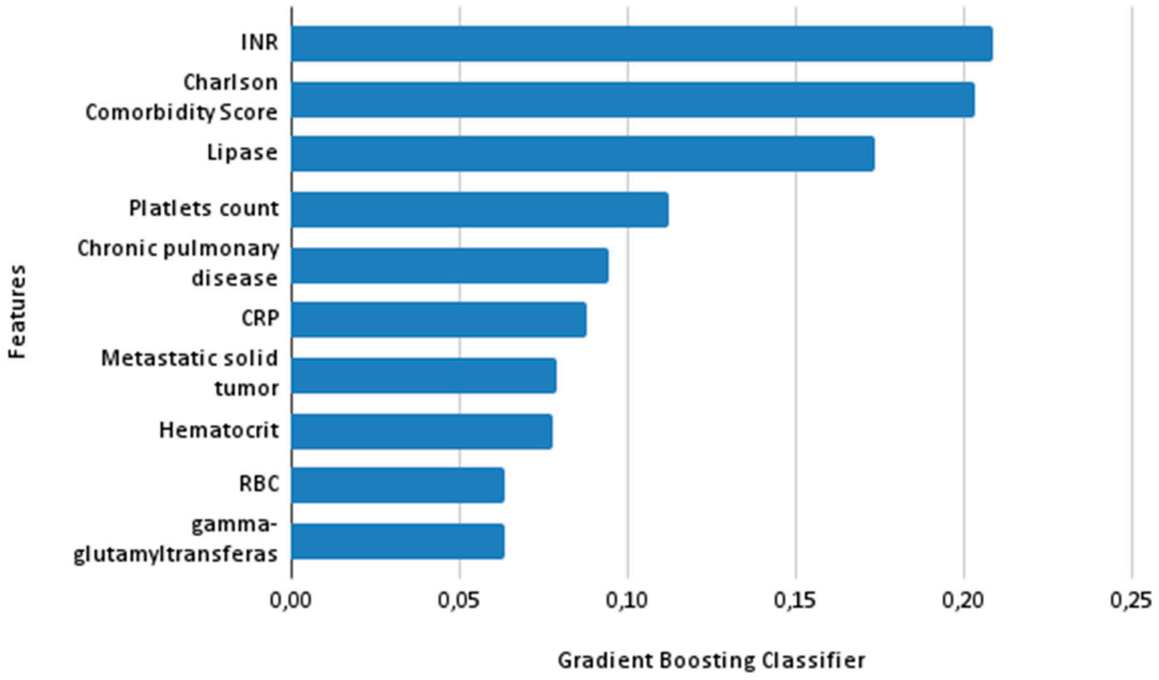

Figure S5: Normalized top ten Shap values Linear SVC, descending.

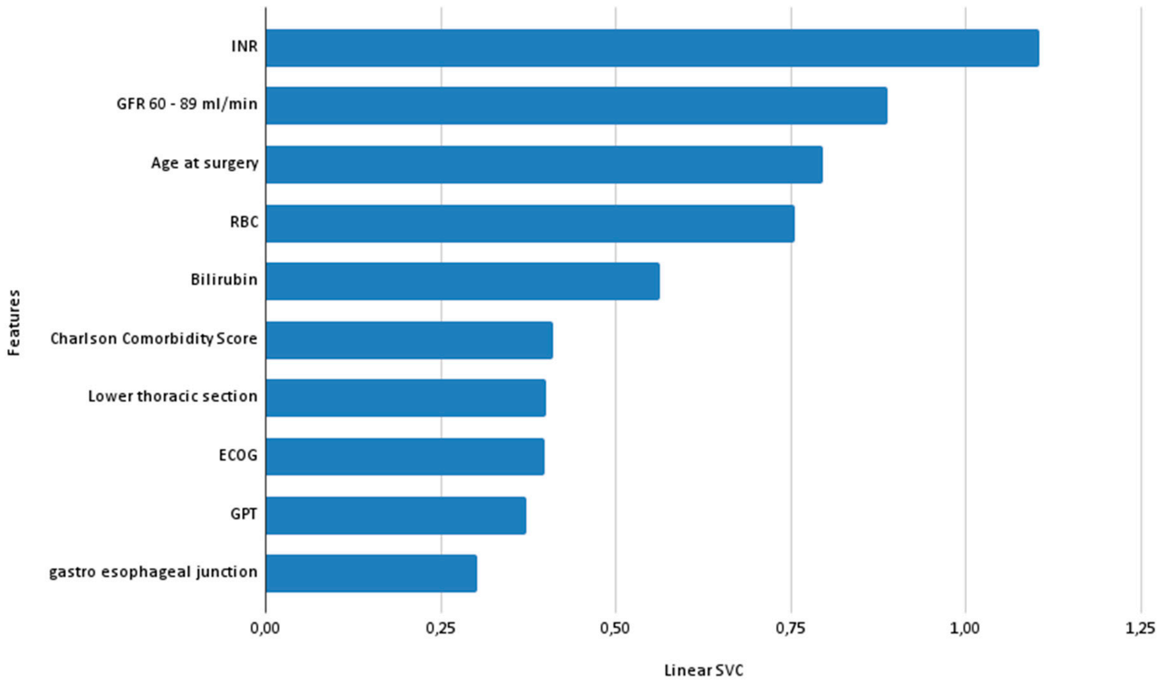

Figure S6: Top ten Shap values Logistic Regression, descending.

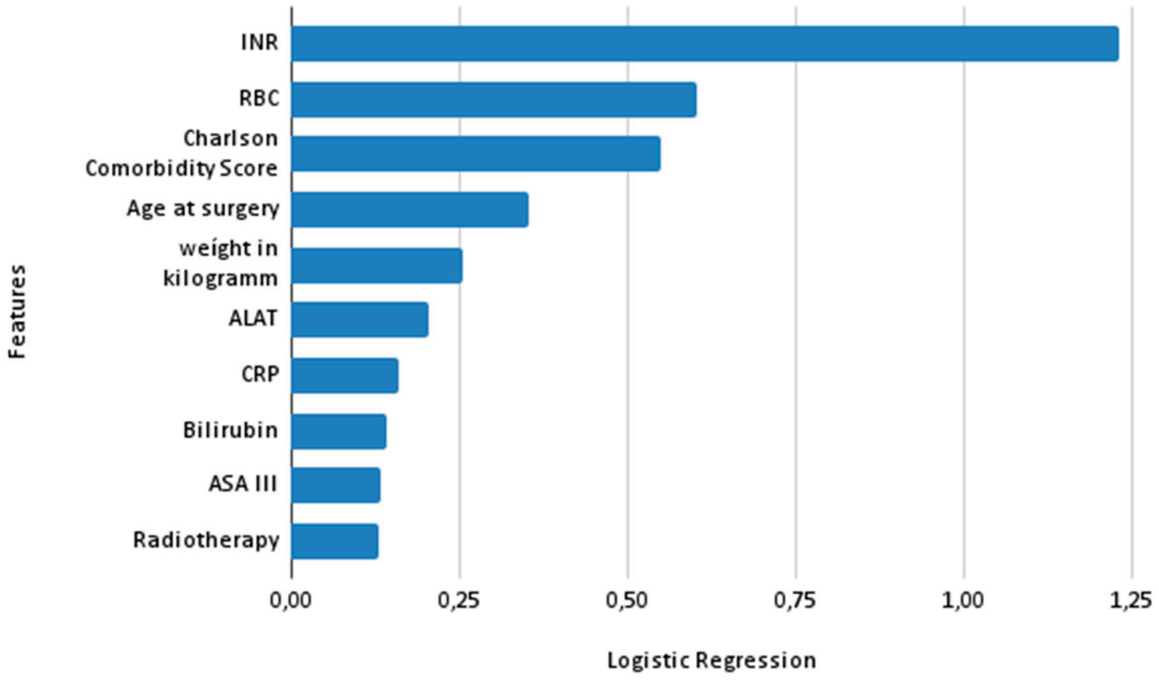

Figure S7: Normalized top ten Shap values Neural Network Regression, descending.

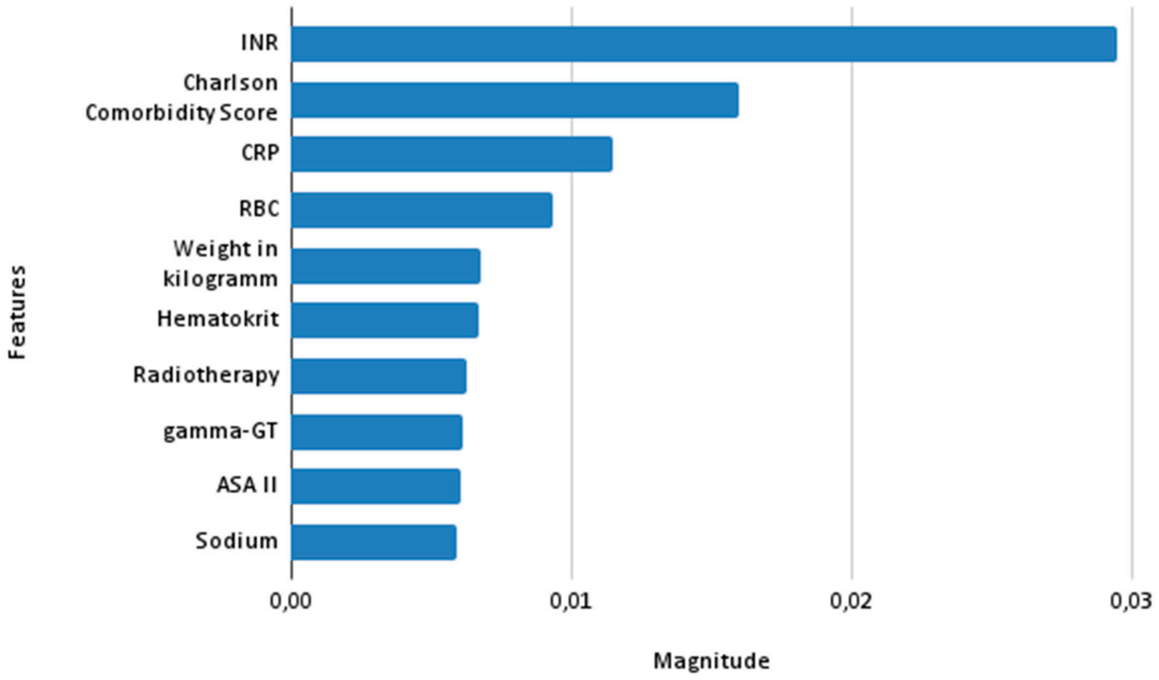

Figure S8: Normalized top ten Shap values Random Forrest Classifier, descending.

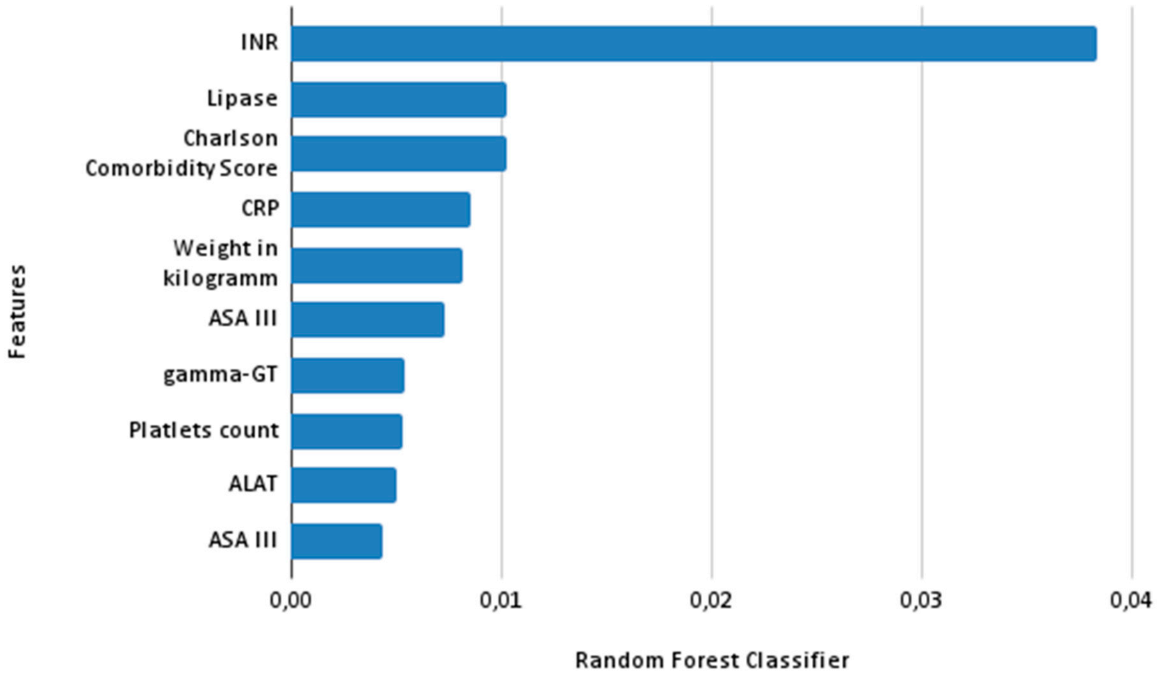

Figure S9: Normalized top ten Shap values Support Vector Classifier, descending.

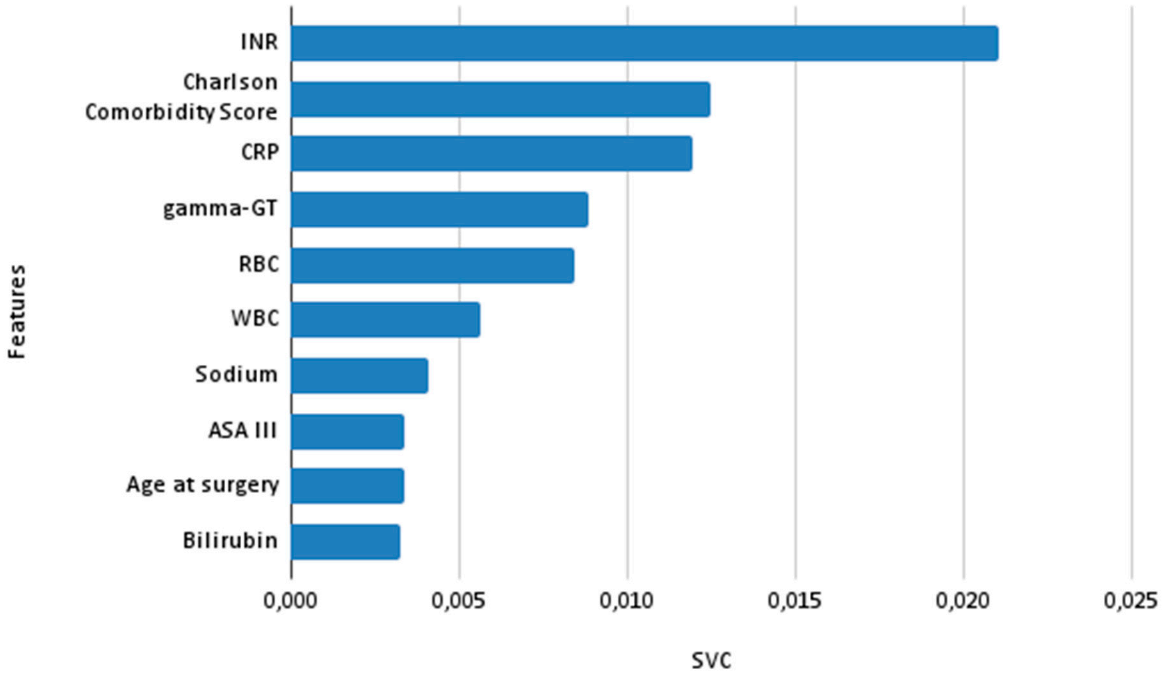

Supplement: Supplementary file 1 [file cancers-16-03000-s001.zip › cancers-3065512-supplementary.pdf]
